# Influence of Implant Location, Number, and Design in Ischemic Zone for Implant Prosthesis Success Rate: A Comparative Three-Dimensional Finite Element Analysis

**DOI:** 10.7759/cureus.53881

**Published:** 2024-02-08

**Authors:** Arshad J Sayed, Kamlesh K Garg, Sasankoti R Mohan, Sabahat Ullah Khan Tareen, Bugude Shiva Shankar, Mostafa H Omran

**Affiliations:** 1 Dentistry, Pacific Academy of Higher Education, Udaipur, IND; 2 Preventive Dentistry (Periodontology), College of Dentistry in Al Rass, Qassim University, Al Rass, SAU; 3 Orthodontics and Dentofacial Orthopedics, Pacific Dental College and Research Centre, Udaipur, IND; 4 Oral Medicine and Radiology, Hope Health Inc, Florence, USA; 5 Operative Dentistry, College of Dentistry in Al Rass, Qassim University, Al Rass, SAU; 6 Oral Hygiene, College of Applied Health Sciences, Qassim University, Al Rass, SAU; 7 Prosthodontics, College of Dentistry in Al Rass, Qassim University, Al Rass, SAU

**Keywords:** implant stability, implant success, stress and strain, finite element analysis, implant prosthesis design, implant dimension

## Abstract

Background: The performance of an implant-supported prosthesis depends on the implant type, number, implant location, and prosthesis design which is directly associated with the distribution of the occlusal forces during mastication. The purpose of the present study is to analyze with three-dimensional (3D) finite element comparative analysis, the influence of implant location, number, and prosthesis design in the mandibular posterior region where multiple posterior teeth replacement is indicated, which in turn is associated with the longevity or Implant success rate.

Material and methods: Mandibular posterior section, where 4 teeth are missing, based on the space available for implants and following the surgical guide instructions, a standard make four implants (1st and 2nd premolars {3.8 mm × 11.5 mm}, 1st and 2nd molar {5.1 mm × 11.5 mm}) were selected and with standardization for placement, 4 groups were created with different implant location, number and prosthesis design from the selected implants as model FM^1^, FM^2^, FM^3^, FM^4^. Finite element analysis was carried out using ANSYS software, version 14.5 (ANSYS Inc., Canonsburg, PA, USA) for assessment of stress, strain, and deformation around implant and bone.

Results: Maximum von Mises stress on vertical loading was highest for FM^4 ^(139.55MPa) model (center of prosthesis on premolar and molar pontics) and lowest for FM^3 ^(53.65MPa) model (on 2nd premolar pontic) with values in decreasing order as FM^4 ^˃ FM^2^ ˃ FM^1^ ˃ FM^3^. Maximum von Mises stress on oblique loading was highest at the distal of 1st molar implant pontic for FM^2 ^(539.81MPa) and lowest at the 2nd premolar pontic for FM^3 ^(352.48MPa) model with values as FM^2^˃FM^1^˃FM^4^˃FM^3^. Deformation for vertical and oblique loading was observed minimum at the buccal cusp and buccal crestal bone of 2nd premolar, 1st molar on FM^3^ model against highest deformation on buccal and lingual crestal bone, cuspal area of 2nd premolar, 1st molar implants. For oblique loading minimum deformation was seen for the 2nd premolar, 1st molar cuspal area in FM^3, ^and maximum at the 2nd premolar region in FM^1^.

Conclusion: Four single implants may be chosen if there is enough mesiodistal and buccolingual space to allow for a minimum inter-implant and inter-implant-tooth distance that can be maintained while putting the least amount of stress on the implants and bone. To reduce stress on the bone and implants, it is best to avoid long-span implant-supported prostheses when using fixed implant-supported prostheses.

## Introduction

Osseointegration occurs when an implant is inserted into the living bone and is the key factor for dental implant stability [1]. This depends on the interlocking/bond formed between living bone and implant material [2]; and, sequentially it depends on several factors such as jaw bone quality and quantity [3,4]; surface composition and structure of the implant used; heat and friction during placement of the implant; initial stability; and forces used (vertical and transversal, compression, tension, shear, and bending moments, mastication and bruxism) during placement and when in use [5].

When replacing more than two missing teeth, especially in the ischemic zone (mandibular posterior region), stability and longevity of a prosthesis are paramount in implant prosthodontics [6]. The performance of an implant-supported prosthesis depends on the implant type, number, implant location, and prosthesis design which is directly associated with the distribution of the occlusal forces during mastication [7,8]. In the long span, multiple missing teeth where the replacement of each missing tooth with an individual implant becomes questionable with respect to mesiodistal available space, bone volume, cost, and longevity of the implants placed. In such cases, implant-supported prosthesis, regardless of the design of the implant and its interaction with the surrounding bone, the prosthesis design plays an important role in avoiding high-stress concentrations in supporting bone [9]. Hence the purpose of the present study is to analyze with 3D finite element comparative analysis, the influence of implant location, number, and prosthesis design in the mandibular posterior region where multiple posterior teeth replacement is indicated, which in turn is associated with the longevity or implant success rate. The objectives were (a) to predict the influence of implant location, number, and prosthesis design in the mandibular posterior region where multiple (≥4) posterior teeth (1st premolar, 2nd premolar, 1st molar, and 2nd molar) replacement is indicated for longevity or success of implant prosthesis, and (b) to analyze stress, strain, and displacement using finite element analysis (FEA) for 4 mandibular posterior teeth prosthesis with different implant location, number and prosthesis design.

A null hypothesis H_0 _was tested, as there is no difference in stress, strain, and displacement of posterior molar implants placed in the mandibular area with respect to implant location, implant number, and prosthesis design for longevity or success of implant prosthesis.

## Materials and methods

CBCT imaging acquisition and processing

Cone-beam computed tomography (CBCT) scans were sourced from the data library at the Dental Clinic of Qassim University. Galileo's Comfort Sirona Dental Systems GmbH, Blenheim, Germany, was used to standardize CBCT scans. The scan parameters were 85 KvP, 21 mAs, and 14 seconds, with a field of view (FOV) of 15 × 12 mm. CBCT image with 4 missing mandibular posterior teeth (1st premolar, 2nd premolar, 1st molar, and 2nd molar) with adequate bone quality and quantity were selected to carry out the analysis. CBCT images were exported as DICOM (digital imaging and communication in medicine) and reconstructed with the BlueSkyPlan (version 3.2, 64-bit) implant planning software (Blue Sky Bio, Libertville, Il, USA) at 1 mm thickness. The DICOM files were transformed from a 2D image to a full 3D solid model, 0.125 mm voxel size slices. A 3D graphic model was generated for the cortical and cancellous bone and saved in stereolithography (STL) format. A solid three-dimensional model was created using Geomagic Design X 2022 (Oqton, Inc., San Francisco, CA, USA), reverse engineering from a CBCT scan of mandibular 3D models.

Virtual implant selection and placement in the model

**Figure 1:**
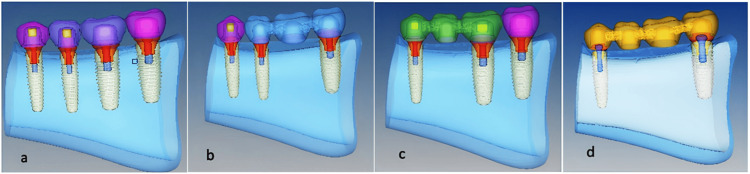
Implant 3D models for replacement of mandibular posterior teeth a. Model FM^1^ (Independent 4 implants; 1st premolar, 2nd premolar, 1st molar, and 2nd molar) b. Model FM^2^ (Independent 1st premolar implant and implant bridge of 2nd premolar to 2nd molar) c. Model FM^3^ (Independent 2nd molar implant and implant bridge of 1st premolar to 1st molar) d. Model FM^4^ (Four-unit implant bridge of 1st premolar to 2nd molar)

Mandibular posterior section, where 4 teeth were missing, based on the space available for implants and following the surgical guide instructions, a standard make four implants (1st and 2nd premolars {3.8 mm × 11.5 mm}, 1st and 2nd molar {5.1 mm × 11.5 mm}) were selected and with standardization for placement, 4 groups were created with different implant location, number and prosthesis design from the selected implants as follows: (a) Model FM^1^: Four individual mandibular implant prostheses (1st premolar, 2nd premolar, 1st molar, and 2nd molar) (Figure 1a); (b) Model FM^2^: An independent 1st premolar implant and implant-supported 3-unit fixed prosthesis with 2nd premolar and 2nd molar abutments with 1st molar pontic (Figure 1b); (c) Model FM^3^: An independent 2nd molar implant and implant-supported 3-unit fixed prosthesis with 1st premolar and 1st molar abutments with 2nd premolar pontic (Figure 1c); (d) Model FM^4^: A 4-unit mandibular implant-supported fixed prosthesis with 1st premolar and 2nd molar as abutments with 2nd premolar and 1st molar pontics (Figure 1d).

The precise geometric data was from the implant library or manufacturer specification, such as length, diameter, and macro-micro thread configuration in millimeters. Using 3-matic software, a CAD model of the dental implant was created.

Finite element analysis model

**Figure 2:**
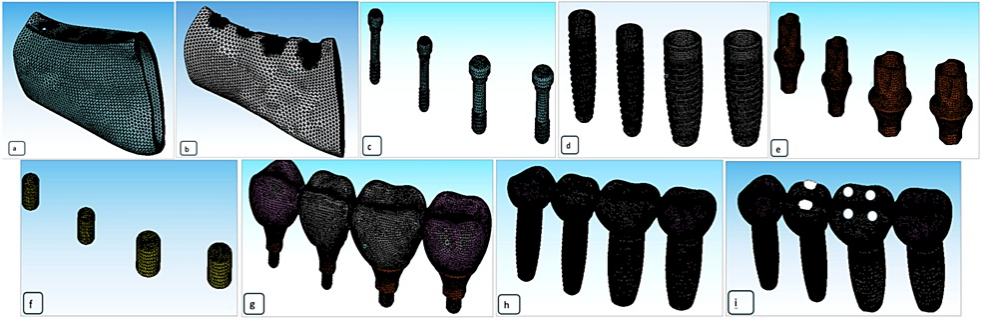
Geometrical modeling and meshing for the required components (a) Mesh of the cortical bone, (b) Mesh of cancellous bone, (c) Mesh of connection screws, (d) Mesh of implant fixtures, (e) Mesh of abutment system, (f) Mesh of abutment component, (g) Mesh of the abutment and crowns, (h) Mesh of crowns and implant system, (i) Loading locations on premolar (2 points contact) and molar (4 points contact) crown

The ANSYS software, version 14.5 (ANSYS Inc., Canonsburg, PA, USA) was used to import the STL files of the mandibular bone section with the prosthesis in situ (all 4 groups) and the properties of the implants and bone (4 FEA models). Mesh creation and geometric modeling for the necessary parts of the models (FM^1^-FM^4^) were simulated, boundary conditions (stress and displacement) were applied, and the results were captured for interpretation (Table 1). The parameters of the material (bone and implant) are included as known from the literature, including the coefficient of friction, Poisson's ratio, and modulus of elasticity [10-15] (Tables 1, 2; Figure 2).

**Table 1:** FEA elements and nodes for implant models FEA: Finite element analysis

Models	Elements	Nodes
Model FM^1^	783431	895273
Model FM^2^	649979	776296
Model FM^3^	650595	778282
Model FM^4^	660118	779119

**Table 2:** Material properties for FEA FEA: Finite element analysis

Materials used	Young’s modulus (MPa)	Poisson’s ratio
Titanium implant	110,000	0.35
Porcelain crown	68,900	0.28
Cortical bone	13,000	0.30
Cancellous bone	1,370	0.30

## Results

The results for the distribution of von Mises stress for an implant-fixed partial denture were analyzed for a number of parameters. They were overall stress, cortical bone stress, cancellous bone stress, implant system stress, connection screw stress, abutment stress, crown system stress, and overall deformation (µm). Vertical loading was 140 N on molars (4-point contacts) and 110 N on premolars (2-point contacts). Oblique loading was 240 N on molars and 100 N on premolars.

Maximum von Mises stress on vertical loading

**Figure 3:**
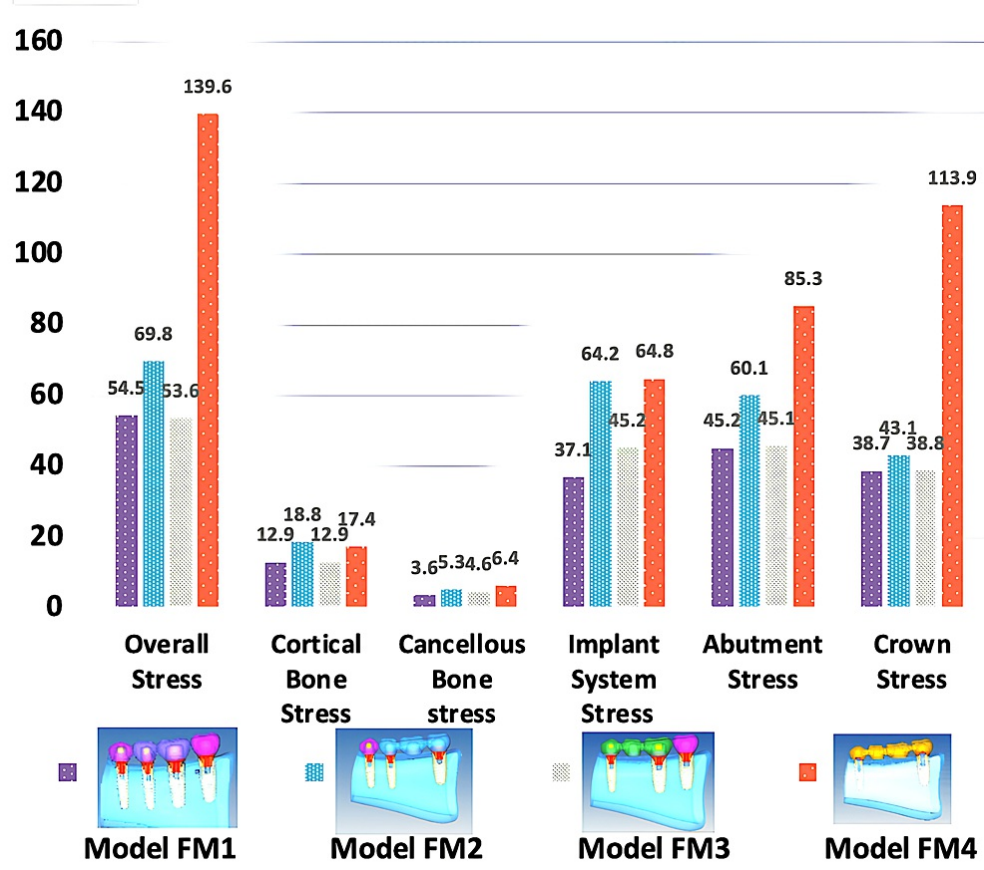
Results of von Mises stress on vertical loading

**Figure 4:**
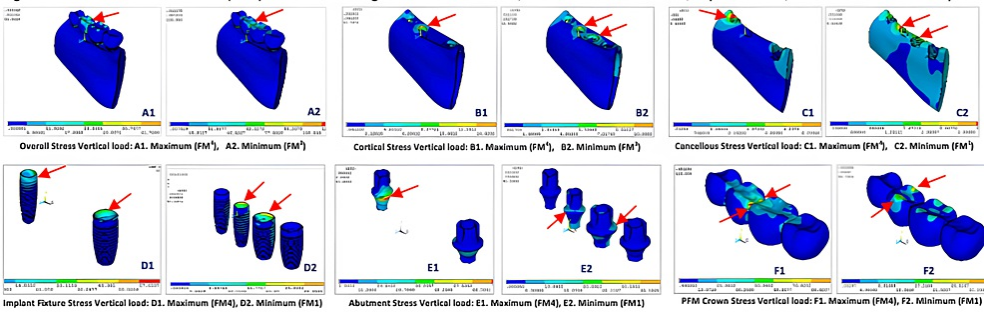
Maximum von Mises stress (MPa) on vertical loading for overall distribution, cortical and cancellous bone, implant fixture, abutment, and PFM crowns PFM crowns: porcelain-fused-to-metal crowns

For vertical loading of 140 N on molars and 110 N on premolars, the results of FEA showed (Figure 3, Table 3) that the overall stress was highest for FM^4^ (139.55MPa) model (center of prosthesis on premolar and molar pontics) and lowest for FM^3^ (53.65MPa) model (on 2nd premolar pontic) with values in decreasing order as FM^4^ ˃ FM^2^ ˃ FM^1^ ˃ FM^3^ (Figure 4; A1, A2).

**Table 3:** Maximum von Mises stress on vertical loading

Vertical load (MPa)	Model FM^1^	Model FM^2^	Model FM^3^	Model FM^4^
Overall stress	54.4707	69.7748	53.6495	139.553
Cortical stress	12.9492	18.7972	12.9717	17.4299
Cancellous stress	3.61409	5.39126	4.63535	6.42996
Implant system stress	37.0971	64.2132	45.2771	64.7971
Abutment stress	45.1993	60.0881	45.8069	85.2886
Crown system stress	38.7385	43.0908	38.7907	113.804

In natural materials such as cortical bone von Mises stress was highest for the FM^2^ (18.80MPa) model (crestal cortical region of 2nd premolar implant) and lowest for FM^1^ (12.95MPa) (crestal cortical region of 2nd premolar implant) with values in order as FM^2^ ˃ FM^4^ ˃ FM^3^ ˃ FM^1^ (Figure 4; B1, B2). Whereas von Mises stress in cancellous bone was highest in FM^4^ (6.43MPa) (mesial aspect of 1st premolar implant at the crestal region) and lowest in FM^1^ (3.61MPa) model (mesial aspect of 2nd premolar implant at the crestal region) with values as FM^4^ ˃ FM^2^ ˃ FM^3^ ˃ FM^1^ (Figure 4; C1, C2). In artificial materials such as implant systems, the von Mises stress was highest at coronal 1/3rd of 1st premolar and crestal tip of 2nd molar in FM^4^ (64.80MPa) and lowest at lingual crestal tip of 2nd premolar and 1st molar implants in FM^1^ (37.09MPa) (FM^4^ ˃ FM^2^ ˃ FM^3^ ˃ FM^1^) (Figure 4; D1, D2), on implant abutment, the von Mises stress was seen highest FM^4^ (85.29MPa) model and lowest in FM^1^ (45.20MPa) model (FM^4^ ˃ FM^2^ ˃ FM^3^ ˃ FM^1^) (Figure 4; E1, E2), and on implant crown system least stress was seen at buccal and lingual cusp tips in FM^1^ (38.74MPa) model (FM^4^ ˃ FM^2^ ˃ FM^3^ ˃ FM^1^) (Figure 4; F1, F2).

Maximum von Mises stress on oblique loading

**Figure 5:**
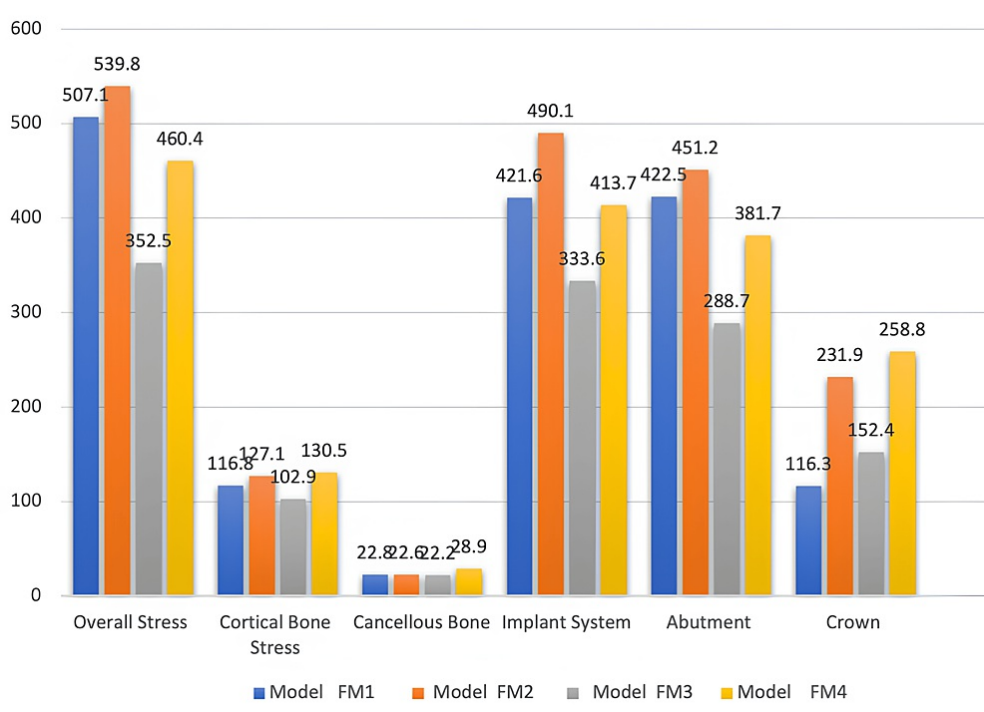
Results of von Mises stress on oblique forces

**Figure 6:**
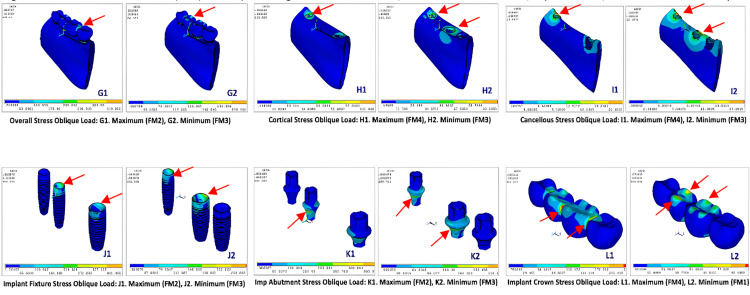
Maximum von Mises stress on oblique loading for overall distribution, cortical and cancellous bone, implant fixture, abutment, and PFM crowns PFM crowns: porcelain-fused-to-metal crowns

When the models were exerted with oblique loading of 240 N on molars and 100 N on premolars, the results of FEA showed (Figure 5, Table 4) that the overall stress distribution was highest at distal of 1st molar implant pontic for FM^2^ (539.81MPa) and lowest at 2nd premolar pontic for FM^3^ (352.48MPa) model with values as FM^2 ^˃ FM^1 ^˃ FM^4 ^˃ FM^3^ (Figure 6; G1, G2).

**Table 4:** Maximum von Mises stress (MPa) on oblique loading

Oblique load (MPa)	Model FM^1^	Model FM^2^	Model FM^3^	Model FM^4^
Overall stress	507.103	539.81	352.484	460.402
Cortical stress	116.787	127.052	102.912	130.508
Cancellous stress	22.77	22.5769	22.1574	28.8577
Implant system stress	421.559	490.136	333.569	413.664
Abutment stress	422.481	451.238	288.714	381.661
Crown system stress	116.281	231.862	152.365	258.827

In cortical bone, von Mises stress was highest at the center of 1st premolar implant for FM^4^ (130.51MPa) and lowest at crestal part of 1st premolar and 1st molar for FM^3^ (102.91MPa) with values in order as FM^4^ ˃ FM^2^ ˃ FM^1^ ˃ FM^3^ (Figure 6; H1, H2). Whereas von Mises stress in cancellous bone was highest at the mesial and buccal surface of 1st premolar area in FM^4^ (28.86MPa) and lowest at coronal portion of 1st premolar and 1st molar region in FM^3^ (22.16MPa) model with values as FM^4^ ˃ FM^1^ ˃ FM^2^ ˃ FM^3^ (Figure 6; I1, I2). In artificial materials such as implant systems, the von Mises stress was highest all around coronal part of 1st premolar implant in FM^2^ (490.14MPa) and lowest at coronal part of 1st premolar and 1st molar implant in FM^3^ (333.57MPa) (FM^2^ ˃ FM^1^ ˃ FM^4^ ˃ FM^3^) (Figure 6; J1, J2), on implant abutment, the von Mises stress was seen highest FM^2^ (451.29MPa) model and lowest in FM^3^ (288.71MPa) model (FM^2^ ˃ FM^1^ ˃ FM^4^ ˃ FM^3^) (Figure 6; K1, K2), and on implant crown system least stress was seen in FM1 (116.28MPa) model with values as (FM^4^ ˃ FM^2^ ˃ FM^3^ ˃ FM^1^) (Figure 6; L1, L2).

Deformation for vertical and oblique loading 

**Figure 7:**
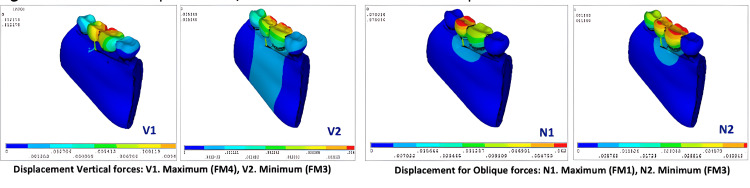
Maximum displacement/deformation on vertical and oblique loading

The overall deformation on vertical loading was observed minimum at the buccal cusp and buccal crestal bone of 2nd premolar, 1st molar on FM^3^ model against highest deformation on buccal and lingual crestal bone, cuspal area of 2nd premolar, 1st molar implants with values as FM^4^ (12.18 µm) ˃ FM^2^ (6.55 µm) ˃ FM^1^ (5.69 µm) ˃ FM^3^ (5.05 µm) (Figure 7; V1, V2) and for oblique loading minimum deformation was seen for 2nd premolar, 1st molar cuspal area in FM^3^ and maximum at 2nd premolar region in FM^1^ with values as FM^1^ (70.40 µm) ˃ FM^2^ (69.75 µm) ˃ FM^4^ (59.51 µm) ˃ FM^3^ (51.87 µm) (Table 5; Figure 7; N1, N2).

**Table 5:** Maximum deformation/displacement on vertical and oblique loading

	Model FM^1^	Model FM^2^	Model FM^3^	Model FM^4^
Overall deformation (µm) for vertical load	5.693	6.553	5.046	12.178
Overall deformation (µm) for oblique load	70.396	69.747	51.869	59.512

## Discussion

Stresses generated in the implant's supra- and sub-structures are distributed to the cancellous and cortical parts of the alveolar bone [16]. Stress concentration of the alveolar crests can significantly affect the longevity of the implant and in turn, the whole of the prosthesis if implants are splinted. Various FEA studies have been conducted to analyze the stress distribution patterns in implants under various loading conditions [17-19]. This FEA study analyzed the stress distribution patterns in implants, implant prosthesis, and alveolar bone under vertical, oblique, and horizontal loading conditions with dental implants in four different configurations (FM^1^, FM^2^, FM^3^, and FM^4^). The occlusal height and occlusal width were uniform in all tested configurations without any cantilever.

In comparison with all other models, model FM^1^ demonstrated the least vertical loading stress value in the implant system, crown, abutment, cancellous bone, and cortical bone. This configuration aligns well with the mechanical design optimization theory and practice. Within the model, the vertical loading stress distribution was highest in the abutment followed by the crown and implant system. Cancellous bone was subjected to the least vertical loading stress followed by connecting screw and cortical bone. This observation in model FM^1^ was in alignment with the observation noted in the Omori M et al. study conducted in 2015 [17]. The implant deformation or implant displacement observations in their study were like the observations in our FEA study. The findings in our FEA study and Omori M et al. study reinforce the concept of clinical consideration of implant replacement for each missing tooth in posterior sextants of the mandible rather than splinting [17]. This gives the highest biomechanical advantage which in turn reflects on stress distribution patterns in implants, prosthetic elements, and alveolar bone. However, such clinical possibilities are determined by limiting factors such as the availability of alveolar bone, alveolar bone configuration, existing occlusion, and patient economic factors.

When masticatory efficiency is considered loss of two premolars can significantly contribute to decreased chewing ability. This emphasizes the importance of replacing the two premolars should be a prime concern in the case of distal extension cases. Replacement of the second molar by implant treatment can have challenges like limited accessibility for surgical procedures and dexterity to use the implant armamentarium during surgery and the prosthetic phase as well. Shao Z et al. in 2018 assessed the associations amongst masticatory performance, dental functional status, and perceived chewing problems in 387 adult participants with reduced natural dentitions [20]. They concluded that in partially dentate people, perceived chewing difficulties were associated with lower objective masticatory efficiency [20]. The two strongest indicators of poor masticatory efficiency were having fewer than 10 teeth in each jaw and having a premolar area that was compromised.

The Zhang Q et al. 2019 study also reinforces the concept of missing two premolars and the decline in masticatory efficiency [21]. Though the study was done in patients with natural teeth, the study results can be extrapolated for clinical decision-making in implant rehabilitation giving due consideration to the occlusal table of two premolars. The higher horizontal loading stress values were associated with model FM^2^ and model FM^1^ in sequence. The least horizontal loading stress value was associated with model FM^3^ and followed by model FM^4^. In a real-time clinical scenario, the major horizontal loading stress will be exerted varyingly by the tongue, buccal musculature, and flexing ability of the mandible. Though our FEA tried to simulate the horizontal loading stress, it was still a limiting factor of our study. When the oblique loading stress distribution pattern was observed in model FM^1^ it revealed that the values are the second highest preceded by model FM^2^. The abutment showed higher oblique loading stress distribution followed by the implant system and crown. This could be attributed to the change in the functional cross-sectional area and the distribution of oblique loading stress distribution. The least oblique loading stress distribution was noted with connecting screw succeeded by cancellous bone and cortical bone.

Model FM^2^ demonstrated the vertical loading stress distribution pattern in second place preceded by model FM^4^. Within the model, the vertical loading stress distribution was highest in the implant system followed by abutment and crown. The oblique loading stress distribution pattern was the highest in model FM^2^ when compared to any other models analyzed in the FEA study. Model FM^1^ and model FM^2^ showed the same deformation values when subjected to horizontal loads. Whereas model FM^3^ demonstrated the least deformation to horizontal loading followed by model FM^4^. Changes in the functional area and the implant configuration are the contributing factors for the varying vertical loading, horizontal loading, and oblique loading stress distribution pattern.

Model FM^4^ demonstrated the highest vertical loading stress value in the implant system, crown, abutment, cancellous bone, and cortical bone. This can be correlated with the configuration model itself as the stress brunt was upon the 1st premolar and 2nd molar. The long-span nature of the implant bridge without any pier abutment in model FM^4^ significantly altered the way the vertical, horizontal, and oblique stresses were distributed in the implant system, abutment, crown, and alveolar bone. According to the conclusion of Rangert et al., the prosthesis, with one or two implant supports, shows a higher tendency for risk of bending and fracture even in 3-unit bridges [22].

Dental implants are subjected to various occlusal loads in various magnitudes, frequencies, and durations depending on the patient’s functional habits. Compressive, tensile, and shear forces are exerted on dental implants in function. Compressive forces tend to maintain the integrity of a bone-implant interface, whereas tensile and shear forces tend to distract or disrupt such an interface. In comparison with other loading modalities, shear forces are most destructive to bone and implants. The cortical bone is weakest in shear and strongest in compression [23]. On occlusal load, a combination of all three types of forces is imparted in threaded dental implants. At the level of designing the implant body, this conversion of three different forces is controlled by implant geometry. For better treatment outcomes and long-term success Compressive forces typically should be dominant in implant prosthetic occlusion.

Mechanical stress is the term used to describe how a force is distributed over a surface. Minimizing the mechanical stress and its even distribution in the implant system and alveolar bone is the key to long-term implant rehabilitation. Force magnitude and cross-sectional area over which the force is distributed are the two variables that determine the magnitude of stress delivered on the implant system and alveolar bone. Crown height, cantilever length and offset loads are the three important magnifiers of force which when controlled can decrease the magnitude of force exerted. However, via meticulous treatment planning, the functional surface area over which the force is dispersed is under control. The functional cross-sectional area can be optimized by increasing the number of implants for a given edentulous site and by choosing an implant geometry that has been meticulously engineered to maximize functional cross-sectional area. The amount of mechanical stress placed on the prosthesis, implant, and biological tissues is reduced when the functional surface area is increased. Deformation of the implant and surrounding tissues occurs when a load is applied to a dental implant system. Within the physiological limits, the biological tissues may be able to cope by remodeling activity.

A two-pontic restoration flexes eight times more than a one-pontic prosthesis, while a three-pontic prosthesis flexes 18 times more than a two-pontic restoration. Increasing the surface area of the implant support system is a vital biomechanical strategy for reducing stress [24]. Increasing the number of implants used to support a prosthesis is the most efficient way to enhance the surface area of implant support. Studies by Bidez and Misch showed that force applied over three abutments causes less localized stress to the crestal bone than does force applied over two abutments [25]. To minimize stress in implant systems and alveolar bone it is always recommended that the number of pontics should be reduced, and the number of implant abutments should be increased [26,27].

## Conclusions

Based on the current study's findings, it can be said that four single implants may be chosen if there is enough mesiodistal and buccolingual space to allow for a minimum inter-implant and inter-implant-tooth distance that can be maintained while putting the least amount of stress on the implants and bone. To reduce stress on the bone and implants, it is best to avoid long span implant supported prostheses when using fixed implant supported prostheses. Instead, small span solutions with many implants are suggested.
